# THREE DECADES OF RESEARCH GUIDING POLICY AND POLICY GUIDING RESEARCH IN AGING AT THE UNIVERSITY OF SOUTH FLORIDA

**DOI:** 10.1093/geroni/igad104.1774

**Published:** 2023-12-21

**Authors:** Lindsay Peterson

**Affiliations:** University of South Florida, Tampa, Florida, United States

## Abstract

The Florida Legislature created the Florida Policy Exchange Center on Aging more than 30 years ago to conduct independent research and policy analyses on critical topics in aging. Housed at the University of South Florida (USF), the center brought together students and faculty to examine public policies that directly affect the health and well-being of older adults (e.g., the role of Medicaid funding in home- and community-based care and the effects of nurse staffing levels in nursing homes). As new policy questions emerge, the foundational work of the policy center continues to guide undergraduate and graduate education at the USF School of Aging Studies. This presentation describes the multiple opportunities to incorporate policy instruction into an interdisciplinary gerontology program. These opportunities depend on having faculty who maintain ties with the organizations that provide direct care and services to the public and who connect the policy lessons they learn through these relationships to their teaching and research. Another key element is access to health services data needed to answer critical policy questions. The goal of such an approach is to enable gerontology students to engage in policy analysis and development and to understand the essential links between research, public policy, and practice that improves the lives of older adults.

